# Obstinate Case of Constipation

**Published:** 1887-08

**Authors:** 


### SURGICAL SOCIETY OF BOURNEVILLE.

(From "*Le Progres Medical*." No. 22.) Translated by Professor Frank Billings.

PROFFESSOR SEE reported the following case: A young man, coming from Salonica to France, was troubled with obstinate constipation during the voyage. On his arrival in France, he underwent various kinds of treatment usual in such cases. Nothing relieved him. He was sent to the "Maison de Santé," where electricty was tried without success. The abdomen was enormously distended and painful, but there was no vomiting, no fever. Water was injected into the intestine in great quantities, to forcibly dilate it, and at the end of four days the patient was cured. (Stools abundant). This case is curious, because the constipation lasted forty-two days. The respiratory troubles, caused by flatulency, were such that it was necessary to tap the intestine, at different times, with a slender trocar in order to allow the gas to escape. Each capillary tapping gave temporary relief, at once.

